# Exogenous pulmonary surfactant for the treatment of adult patients with acute respiratory distress syndrome: results of a meta-analysis

**DOI:** 10.1186/cc4851

**Published:** 2006-03-08

**Authors:** Warren J Davidson, Del Dorscheid, Roger Spragg, Michael Schulzer, Edwin Mak, Najib T Ayas

**Affiliations:** 1 Department of Medicine University of British Columbia, Vancouver, British Columbia, Canada; 2 Intensive Care Unit Providence Healthcare, Vancouver, British Columbia, Canada; 3 University of California at San Diego, California, USA; 4 Centre for Clinical Epidemiology and Evaluation, Vancouver Coastal Health Research Institute, Vancouver, British Columbia, Canada

## Abstract

**Introduction:**

The purpose of this study was to perform a systematic review and meta-analysis of exogenous surfactant administration to assess whether this therapy may be useful in adult patients with acute respiratory distress syndrome.

**Methods:**

We performed a computerized literature search from 1966 to December 2005 to identify randomized clinical trials. The primary outcome measure was mortality 28–30 days after randomization. Secondary outcome measures included a change in oxygenation (PaO_2_:FiO_2 _ratio), the number of ventilation-free days, and the mean duration of ventilation. Meta-analysis was performed using the inverse variance method.

**Results:**

Two hundred and fifty-one articles were identified. Five studies met our inclusion criteria. Treatment with pulmonary surfactant was not associated with reduced mortality compared with the control group (odds ratio 0.97; 95% confidence interval (CI) 0.73, 1.30). Subgroup analysis revealed no difference between surfactant containing surface protein or not – the pooled odds ratio for mortality was 0.87 (95% CI 0.48, 1.58) for trials using surface protein and the odds ratio was 1.08 (95% CI 0.72, 1.64) for trials without surface protein. The mean difference in change in the PaO_2_:FiO_2 _ratio was not significant (*P *= 0.11). There was a trend for improved oxygenation in the surfactant group (pooled mean change 13.18 mmHg, standard error 8.23 mmHg; 95% CI -2.95, 29.32). The number of ventilation-free days and the mean duration of ventilation could not undergo pooled analysis due to a lack of sufficient data.

**Conclusion:**

Exogenous surfactant may improve oxygenation but has not been shown to improve mortality. Currently, exogenous surfactant cannot be considered an effective adjunctive therapy in acute respiratory distress syndrome.

## Introduction

Acute respiratory distress syndrome (ARDS) is a common cause of respiratory failure in the intensive care unit. Patients with ARDS exhibit an intense inflammatory reaction centered in the lung parenchyma, resulting in alveolar flooding and collapse, in reduced lung compliance, in increased work of breathing, and in severe impairments in gas exchange [1-4]. Patients with ARDS have an inhospital mortality rate ranging from 34% to 60% [5]. Treatment of patients with ARDS is largely supportive, and includes mechanical ventilation with low tidal volumes [6], positive end expiratory pressure to open collapsed alveoli [7], supplemental oxygen, and supportive care of other organ system failures. Given the high mortality rate of patients with ARDS, other therapies are clearly needed.

Administration of exogenous pulmonary surfactant is an adjunctive therapy that may help adult patients with ARDS. Pulmonary surfactant is produced by type II alveolar cells and is composed of two major fractions: phospholipids (90%) and surfactant-specific proteins (10%). Surfactant decreases alveolar surface tension, thereby preventing alveolar collapse and allowing efficient gas exchange at low transpulmonary pressures. Furthermore, surfactant has important roles in host immune defense, through both specific and nonspecific mechanisms [8].

Patients with ARDS show injury to the alveolar epithelial barrier with consequent surfactant dysfunction. Indeed, surfactant recovered from bronchoalveolar lavage fluid from ARDS patients has alterations of the phospholipid and fatty acid profile, has decreased levels of surfactant-specific proteins, and has impaired surface-tension-lowering properties. Causes of this impairment include the inhibition of surfactant function by protein-rich edema fluid, by surfactant lipid peroxidation, and by surfactant protein degradation [1,9]. Given these abnormalities, administration of exogenous pulmonary surfactant has been considered a possible treatment option in adult patients with ARDS [8].

The purpose of this study was to perform a systematic review and meta-analysis of exogenous surfactant administration to assess whether this therapy, as currently administered, may be useful in adult patients with ARDS.

## Materials and methods

### Study identification

We performed a computerized search to identify articles that compared treatment with exogenous pulmonary surfactant against the usual therapy for patients diagnosed with ARDS. For our analysis, we only included studies that were randomized controlled clinical trials, that compared the use of exogenous pulmonary surfactant to an appropriate control group (defined as patients receiving standard therapy with or without a placebo), that evaluated mortality and/or pulmonary physiological parameters, and that used objective documentation of ARDS using accepted criteria at the time of the individual study publication. Abstracts, case reports, editorials, nonhuman studies, and nonEnglish studies were excluded.

We performed a computerized literature search of MEDLINE (1966–December 2005), EMBASE (1980–December 2005), Cochrane Database of Systematic Reviews (1996–December 2005), Cochrane controlled trials register (1996–December 2005), and the Database of Abstracts and Reviews of Effects (1994–December 2005) to identify clinical studies and systematic reviews. We conducted the search for human studies using the following combination of exploded medical subject headings and text words: ('adult respiratory distress syndrome' or 'acute respiratory distress syndrome' or 'ARDS') and ('pulmonary surfactant' or 'lung surfactant') and ('adult'). The reference lists of all articles selected were then hand-searched for additional citations missed in the search.

### Study selection

Two authors (WJD, NTA) independently reviewed the abstracts of the references identified to determine suitability for inclusion. Studies that could potentially be included were obtained and reviewed in detail. Examiners were not blinded to authors, to institutions, or to journal name.

### Data extraction

Information about relevant outcome measures was extracted for each study. Our primary outcome measure was mortality 28–30 days after randomization. Secondary outcome measures included a change in oxygenation (specifically the change in the ratio between the partial pressure of oxygen and the fraction of inspired oxygen (PaO_2_:FiO_2 _ratio)), the number of ventilation-free days, and the mean duration of ventilation. Furthermore, the following data were extracted: method of randomization; inclusion and exclusion criteria; details of surfactant administration, including type of surfactant, dose, duration, and delivery method; nature of control treatment; mean age or age range; gender ratio; ARDS scoring system; etiologies of ARDS; and ventilation strategy.

Methodologic quality was assessed using the Jadad scoring system, which consists of items describing randomization (0–2 points), blinding (0–2 points), and dropouts and withdrawals (0–1 points) in reporting of a randomized controlled trial [10]. A higher score indicates improved reporting. One author (WJD) extracted the data, which were reviewed by the two other authors (NTA, DD). If disagreement occurred, all three authors met to establish consensus. If relevant data were missing or unclear from a particular trial, we attempted to contact the primary author of that study.

### Statistical analysis

Meta-analysis was performed using the inverse variance method. Statistical heterogeneity was evaluated using the Q statistic with *P *< 0.1. The primary outcome was summarized as the odds ratio (OR) with the 95% confidence interval (CI). A fixed-effect model was used unless there was significant heterogeneity, in which case we applied a random effects model. We examined the influence of the method of delivery and the type of surfactant on all trials using predetermined sensitivity analyses. All statistical analyses were performed using Stata Version 8.0 (Statacorp LP, College Station, Texas, USA).

### Ethics

Ethics approval and patient consent were not applicable for this meta-analysis.

## Results

### Search Results

We initially identified 251 articles. Of these, we excluded 238 because titles or abstracts were not relevant. Thirteen studies were retrieved for detailed review [11-23]. Four studies were added from a hand search of articles and clinical trials [24-27]. Twelve studies were not eligible for analysis (Table 1): seven were in abstract form only [21-27], four had no control group [11-13,27], and one was a crossover trial [15]. Five studies met our inclusion criteria (Table 2) [16-20]. The study by Spragg and colleagues [20] included results from both a North American trial and a European–South African trial. For the purposes of our analysis, therefore, the data from the two trials in this manuscript were assessed independently, resulting in the final analysis of data from six randomized controlled trials [16-20].

### Study characteristics

The studies were published from November 1994 to August 2004 (Table 2). All were multicenter trials. The number of patients in each trial ranged from 39 to 725. Different doses of surfactant were used in three trials [16,18,19].

In an effort to analyze the most comparable data, the surfactant group in the study by Weg and colleagues [16] with the closest dosing to the surfactant group in the study by Anzueto and colleagues [17] was chosen for analysis. This resulted in the exclusion of 17 patients.

A similar issue was found in the four trials using surfactant containing surface protein. Specifically, in the trial by Spragg and colleagues [19] the surfactant group chosen for analysis was the group who were given the same dose of surfactant as the two other trials [20] using the same type of surfactant (recombinant surface protein C). This resulted in the exclusion of 12 patients. In the trial by Gregory and colleagues [18] the group that received the higher dose of surfactant was used for analysis. As a result, 24 patients were excluded from the analysis.

A total of 1,270 patients were analyzed in these six trials: 381 patients were given surfactant containing no surfactant protein (two trials) [16,17]; 239 patients were given surfactant containing recombinant surfactant protein C (three trials) [19,20]; and 19 patients were given bovine surfactant containing both surfactant proteins B and C (one trial) [18].

All studies included ARDS resulting from sepsis. Two studies only included patients with sepsis-related ARDS, both pulmonary and nonpulmonary [16,17]. The remaining studies included patients with other direct lung injury (aspiration) and indirect lung injury (trauma or surgery, transfusions, pancreatitis, burns, and toxic injury).

### Primary outcome (mortality at 28 or 30 days)

**Figure 1:**
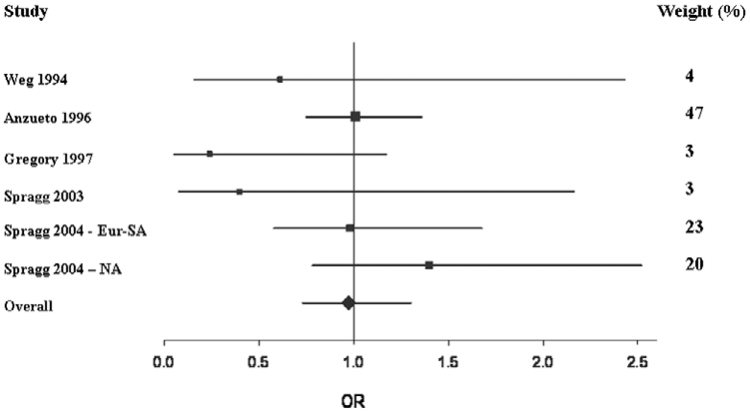
Forest plot of mortality. This Forest plot represents the odds ratio (OR) (95% confidence interval) for 28-day to 30-day mortality in patients treated with surfactant compared with controls. OR < 1 indicates that treatment with surfactant was associated with a reduction in mortality compared with the control group, while OR > 1 indicates an increase in mortality with surfactant therapy. Areas of boxes are proportional to the respective study weight within the corresponding pooled analysis (see also weight values on the right). Eur-SA, European–South African trial; NA, North American trial.

Overall, treatment with exogenous pulmonary surfactant was not associated with reduced mortality compared with the control group (Figure 1 and Table 3). That is, compared with the control group, the OR for mortality after treatment with surfactant was 0.97 (95% CI 0.73, 1.30). Subgroup analysis revealed no difference between the aerosolized and intratracheal instillation methods: OR 0.99 (95% CI 0.74, 1.32) and 0.87 (95% CI 0.48, 1.58), respectively (Table 3).

Furthermore, the OR for mortality was similar regardless of whether the surfactant contained surface protein or not. That is, the pooled OR for mortality was 1.08 (95% CI 0.72, 1.64) for the two trials using surfactant without surface protein [16,17], and was 0.87 (95% CI 0.48, 1.58) for the four trials using surfactant containing surface protein B and/or protein C [18-20] (Table 3).

### Secondary outcomes

**Figure 2:**
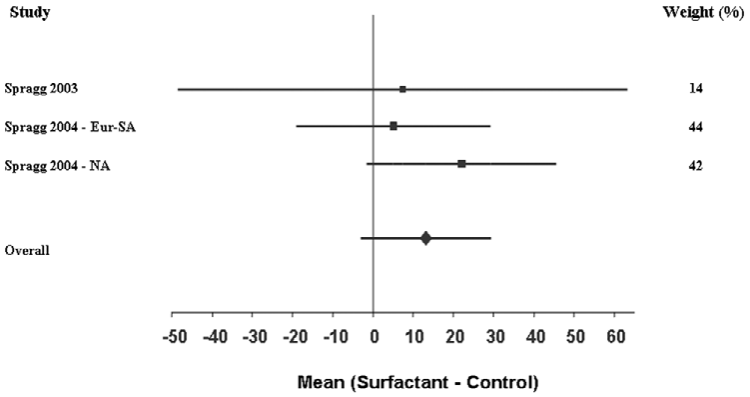
Forest plot of the PaO_2_:FiO_2 _ratio. This Forest plot represents the mean difference in the change in the PaO_2_:FiO_2 _ratio (mmHg) of surfactant compared with controls. A positive value (i.e. right of 0) indicates that treatment with surfactant resulted in improved oxygenation at 24 hours compared with controls. Areas of boxes are proportional to the respective study weight within the corresponding pooled analysis (see also weight values on the right). Eur-SA, European–South African trial; NA, North American trial.

Due to the constraints of the published data, the mean difference in change in the PaO_2_:FiO_2 _ratio between the surfactant and control groups could only be assessed at the 24-hour mark following treatment administration. Three studies had sufficient information to allow pooling of the PaO_2_:FiO_2 _data [19,20]. These three trials studied a total of 488 patients (251 patients in the surfactant arm and 237 patients in the control arm). A fixed-effect model was used because the Q test for heterogeneity was not significant (*P *= 0.11). There was a trend for the surfactant group to have improved oxygenation compared with the controls. This did not achieve statistical significance, however (pooled mean change 13.18 mmHg, standard error 8.23 mmHg; 95% CI -2.95, 29.32) (Figure 2).

The number of ventilation-free days and the mean duration of ventilation could not undergo pooled analysis due to a lack of sufficient data.

## Discussion

Adult patients with ARDS exhibit a reduction in the amount and function of surface-active material recovered by bronchoalveolar lavage. In addition, the phospholipid, fatty acid, and apoprotein profiles of pulmonary surfactant are altered [1]. It would therefore seem sensible that exogenous pulmonary surfactant would be a useful therapy in the treatment of ARDS. Our meta-analysis of six randomized controlled trials, however, demonstrated little utility of the therapy [16-20]. There was no overall improvement in mortality (OR 0.97; 95% CI 0.73, 1.30). Furthermore, subgroup analysis of preparations with surfactant proteins in addition to phospholipids did not demonstrate improved outcomes (OR 0.87; 95% CI 0.48, 1.58). In three of the studies we were able to assess the impact of surfactant on oxygenation (for instance the PaO_2_:FiO_2 _ratio 24 hours following surfactant administration). Although there was a trend to improved oxygenation, this did not reach statistical significance (mean change 13.18 mmHg, standard error 8.23 mmHg; 95% CI -2.95, 29.32).

Our search for all published randomized controlled trials was thorough. Each study was assessed for quality and was chosen only if they were similar with respect to study participants and outcome measure. Mortality was chosen as the primary outcome given its importance in clinical practice. Unlike the most recent published meta-analysis [28], we attempted to assess oxygenation (PaO_2_:FiO_2 _ratio), the number of ventilation-free days, and the mean duration of ventilation. Unfortunately, there were limited data available for analysis of the change in oxygenation and insufficient data for assessment of ventilation characteristics. It is possible that we may have missed some published and unpublished articles.

The quality of the studies varied in our meta-analysis. Using the Jadad scoring system [10], four of the studies were of high quality (Jadad score 4 or 5) [16,17,20] but two studies were not (Jadad score 2) [18,19] (Table 4). Of the latter two studies, one was a phase I/II prospective, randomized trial while the other was open-labeled. Notably these two studies had the lowest OR for mortality, and their exclusion, which would favor the null hypothesis, would not have changed our results significantly.

A limitation of our analysis is the many differences among the various studies. First, different types of surfactant were used. Two of the studies used synthetic surfactant (Exosurf) containing no surfactant protein [16,17]. These studies have been criticized given the emerging data on the importance of surfactant proteins in the proper functioning of surfactant [29,30]. It has been shown that surfactant-associated protein concentrations are decreased in bronchoalveolar lavage samples obtained from patients with ARDS compared with samples from control subjects [3]. Four surfactant proteins have been previously identified (SP-A, SP-B, SP-C, and SP-D). SP-B and SP-C are hydrophobic proteins that enhance the lowering of surface tension [8]. In the three studies using protein-based surfactant, two were recombinant preparations incorporating SP-C [19,20] while the other was a bovine extract with both SP-B and SP-C [18]. SP-A and SP-D are hydrophilic proteins whose role appears to center around host defense [8]. None of the trials in our analysis, however, used surfactant containing SP-A or SP-D. It is possible that the presence of these proteins could increase the effectiveness of therapy.

Second, the different delivery methods used may have resulted in varying concentrations of surfactant reaching the damaged alveoli and altering the effectiveness of therapy. It has been shown that the relative rate of pulmonary deposition of surfactant is 4–5% using the aerosolization route [17,29,30]. In the article by Anzueto and colleagues [17] this would correspond to delivery of less than 5 mg/kg/day phospholipid, while other investigations have suggested that administration of 300 mg/kg/day may be required [30]. The ability of intratracheal administration, the method used by most of the studies in this meta-analysis, to effectively deliver of surfactant to the alveoli is unclear. Delivery of surfactant using the bronchoscopic route has been shown to be efficacious and safe, with initial studies showing improved oxygenation and a trend toward improved mortality [11-15]. None of the trials using this method, however, met the inclusion criteria for our analysis. Nevertheless, bronchoscopic administration may be a potential promising path of future investigation.

Third, there were a variety of other differences between the studies including ventilation strategies and the time to surfactant administration. In this meta-analysis, three studies utilized the low tidal volume approach [19,20] while one trial used traditional tidal volumes [18]. Two trials did not specify the ventilation strategy used [16,17]. Most studies required administration of surfactant within 48 hours of the diagnosis of ARDS. One study allowed administration up to 72 hours after ARDS was diagnosed [20]. The timing of administration is an important issue as the response to early therapy versus delayed therapy may be significant [3].

Finally, the populations that were studied included patients with a wide variety of predisposing causes for ARDS. Patients with ARDS associated with indirect causes, for example sepsis, trauma, or pancreatitis, have a greater number of potentially fatal comorbidities than do patients with ARDS from direct causes such as aspiration or pneumonia [20]. Surfactant is unlikely to prevent nonpulmonary causes of death, and thus may only be effective in the subset of ARDS patients with direct lung injury. In a recent study of pediatric patients with acute lung injury, treatment with surfactant significantly improved oxygenation and survival in the subgroup of patients with direct acute lung injury, while having little effect on patients with indirect acute lung injury [31]. To date, studies focusing on the adult population with direct acute lung injury have not been reported.

Our results confirm and extend those of Adhikari and colleagues [28], who recently published a meta-analysis of a variety pharmacologic agents (for instance prostaglandin E, *N*-acetylcysteine, high-dose steroids, pulmonary surfactant, pentoxifylline) used in the treatment of ARDS and acute lung injury. Their review had significant differences compared with ours, however. First, five of the nine studies included in their review were abstracts, several of which did not include a placebo group. Second, they were only able to assess early mortality and did not include the change in the PaO_2_:FiO_2 _ratio. Finally, they did not perform subgroup analyses. Despite these methodologic differences, their results were consistent with ours in that exogenous pulmonary surfactant was found to have no significant effect on mortality (relative risk 0.93; 95% CI 0.77, 1.12).

## Conclusion

We found in our meta-analysis that exogenous surfactant may improve oxygenation but did not improve mortality. Given the abnormalities of surfactant function found in patients with ARDS, the lack of effectiveness of exogenous surfactant is somewhat surprising. One potential explanation is that patients with ARDS usually die of multi-organ system failure from their underlying disease process (for example sepsis) rather than from respiratory failure *per se*. As such, treatment of the pulmonary abnormalities may not affect mortality substantially. Evaluation of surfactant treatment of patients with direct lung injury may clarify this issue. Another potential explanation is that the proper 'surfactant recipe' has not yet been found. That is, there may be a dose, formulation, and delivery strategy of surfactant that could be effective in patients in ARDS, potentially when combined with other therapies such as lung protective ventilation [6], high-frequency ventilation [32], prone positioning [33], or extracorporeal membrane oxygenation [34]. Future studies may eventually discover such an approach, but exogenous surfactant cannot currently be considered an effective adjunctive therapy in ARDS.

## Key messages

• 	Exogenous pulmonary surfactant may improve oxygenation in patients with ARDS.

• 	Exogenous pulmonary surfactant cannot currently be considered an effective adjunctive therapy in patients with ARDS.

## Abbreviations

ARDS = acute respiratory distress syndrome; CI = confidence interval; FiO_2 _= fraction of inspired oxygen; OR = odds ratio; PaO_2 _= partial pressure of oxygen in arterial blood.

## Competing interests

Roger Spragg serves as a consultant to Altana. Najib Ayas is supported by a Scholar Award from the Michael Smith Foundation for Health Research, a New Investigator Award from the BC Lung Association and CIHR, and a Departmental Scholar Award from the University of British Columbia. Del Dorscheid is supported by a Scholar Award from the Michael Smith Foundation for Health Research, operating grants from BC Lung Association, Canadian Institutes of Health Research, and the National Institutes of Health (NIH 66026).

## Authors' contributions

WJD conceived of the study, participated in its design and coordination, and helped to draft the manuscript. DD, RS, and NTA participated in the study design and helped to draft the manuscript. MS and EM performed the statistical analysis. All authors read and approved the final manuscript.

## Figures and Tables

**Table 1:** Characteristics of the trials not eligible for meta-analysis

Reference	Number of patients	Exclusion criteria	Delivery method	Type of surfactant	Other remarks
Reines and colleagues, 1992 [27]	49	Abstract only	Aerosolized	Exosurf (synthetic, no surfactant protein)	Published as an abstract. Placebo-controlled. Trend for improvement in the PaO_2_:FiO_2 _ratio and mortality
MacIntyre and colleagues, 1994 [26]	10	Abstract only. No control group. No data on oxygenation or mortality	Aerosolized	Exosurf (synthetic, no surfactant protein)	Published as an abstract. Only 4.5% of aerosolized radiolabeled surfactant reached the lungs
Spragg and colleagues, 1994 [15]	6	Crossover trial	Bronchoscopic	Porcine surfactant	Trend for improved oxygenation. Findings of reduced inhibition of surfactant function in bronchoalveolar lavage fluid after surfactant replacement
Walmrath and colleagues, 1996 [13]	10	No control group	Bronchoscopic	Alveofact (natural bovine surfactant)	Trend for improvement in oxygenation (PaO_2_:FiO_2 _ratio)
Pallua and colleagues, 1998 [12]	4	No control group	Bronchoscopic	Alveofact (natural bovine surfactant)	Improved oxygenation (PaO_2_:FiO_2 _ratio)
Wiswell and colleagues, 1999 [11]	12	No control group	Bronchoscopic	Surfaxin (synthetic surfactant)	Surfactant administration was safe. FiO_2 _and positive end-expiratory pressure decreased after treatment initiation
Walmrath and colleagues, 2000 [25]	41	Abstract only	Intratracheal	Venticute (rSP-C-based surfactant)	Published as an abstract. Randomized. Trend for improvement in PaO_2_:FiO_2 _ratio, number of ventilator-free days and successful weaning at 28 days in patients receiving surfactant
Kesecioglu and colleagues, 2001 [22]	36	Abstract only	Intratracheal	Porcine surfactant	Published as an abstract. Randomized. Surfactant administration was safe. PaO_2_:FiO_2 _ratio and survival were improved in surfactant group
Spragg and colleagues, 2001 [24]	40	Abstract only	Intratracheal	Venticute (rSP-C-based surfactant)	Published as an abstract. Randomized. Surfactant treatment may reduce acute pulmonary inflammation
Walmrath and colleagues, 2002 [14]	27	No control group	Bronchoscopic	Alveofact (natural bovine surfactant)	Surfactant administration was safe. Improved PaO_2_:FiO_2 _ratio
Spragg and colleagues, 2002 [23]	448	Abstract only	Intratracheal	Venticute (rSP-C-based surfactant)	Published as an abstract. Randomized. Improved PaO_2_:FiO_2 _ratio. No mortality benefit
Gregory and colleagues, 2003 [21]	22	Abstract only. No control group	Bronchoscopic	Surfaxin (synthetic surfactant)	Published as an abstract. Procedure found to be safe and tolerable

rSP-C, recombinant surfactant protein C.

**Table 2:** Characteristics of the trials eligible for meta-analysis

Article (Jadad score)	Design	Number of patients	Delivery method	Type of surfactant	Surfactant dosing (total)	Treatment duration	Number of deaths		Ventilation-free days^a^		Duration of ventilation^b^	
							
							Control	Surfactant	Control	Surfactant	Control	Surfactant
Weg and colleagues, 1994 [16] (score 5)	Multicenter: USA, Canada	51 (control = 17, group 1 = 17, group 2 = 17)	Aerosolized	Exosurf (synthetic, no surfactant protein)	13.5 mg DPPC/ml (group 1, 21.9 mg DPPC/kg/day; Group 2, 43.5 mg DPPC/kg/day)	Maximum 120 hours for all groups	8	Group 1 = 7, group 2 = 6	NA	NA	NA	NA
Anzueto and colleagues, 1996 [17] (score 5)	Multicenter: USA, Spain, France	725 (control = 361, surfactant = 364)	Aerosolized	Exosurf (synthetic, no surfactant protein)	13.5 mg DPPC/ml (112 mg DPPC/kg/day)	Maximum 5 days	143	145	NA	NA	16.4 (0.9)	16.0 (1.0)
Gregory and colleagues, 1997 [18] (score 2)	Multicenter: USA	59 (control = 16, group 1 = 8, group 2 = 16, group 3 = 19)	Intratracheal	Survanta bovine lung extract (containing SP-B and SP-C)	Group 1, 50 mg/kg LBW (maximum 8 doses); group 2, 100 mg/kg LBW (maximum 4 doses); group 3, 100 mg/kg LBW (maximum 8 doses)	Maximum 96 hours for all groups	7	Group 1 = 4, group 2 = 3, group 3 = 3	NA	NA	10	Group 1 = 15^c^, group 2 = 7^c^, group 3 = 10^c^
Spragg and colleagues, 2003 [19] (score 2)	Multicenter: USA, Canada	40 (control= 13, group 1 = 15, group 2 = 12)	Intratracheal	Venticute (rSP-C-based surfactant)	Group 1, 1 mg/kg LBW (maximum 4 doses); group 2, 0.5 ml/kg LBW (maximum 4 doses)	24 hours for all groups	5	Group 1 = 3, group 2 = 4	6 (0–15)	Group 1= 5 (0–18), group 2 = 4 (0–12)	NA	NA
Spragg and colleagues, 2004 [20] (score 4)	Multicenter: Europe, South Africa	227 (control = 109, surfactant = 118)	Intratracheal	rSP-C-based surfactant	1 mg/kg LBW (maximum 4 doses)	24 hours	43	46	0 (0–20)	0 (0–19)	NA	NA
Spragg and colleagues, 2004 [20] (score 4)	Multicenter: USA, Canada	221 (control = 115, surfactant = 106)	Intratracheal	rSP-C-based surfactant	1 mg/kg LBW (maximum 4 doses)	24 hours	29	34	6 (0–21)	3.5 (0–21)	NA	NA

DPPC, dipalmitoylphosphatidylcholine; LBW, lean body weight; rSP-C, recombinant surfactant protein C; NA, not available.

^a^Values presented as median (25th–75th percentile).

^b^Values presented as mean (± standard deviation).

^c^Values presented as median.

**Table 3:** Principal outcome measures in patients according to type of surfactant and method of delivery

Outcome	Number of trials	Number of patients		Heterogeneity		*P *value	Fixed-effects model [odds ratio (95% CI)]	Random-effects model [odds ratio (95% CI)]
					
		Control	Surfactant^a^	Q statistic	*I*^2^			
Overall mortality	6	631	639	6.00	0.17	0.31	0.99 (0.79, 1.25)	0.97 (0.73, 1.30)
Method of delivery								
Aerosolized [16,17]	2	378	381	0.48	0	0.49	0.99 (0.74, 1.32)	-
Intratracheal [18-20]	4	253	258	5.52	0.46	0.14	1.00 (0.69, 1.46)	0.87 (0.48, 1.58)
Type of surfactant								
Synthetic [16,17] (no surfactant protein)	2	378	381	2.21	0.55	0.14	1.09 (0.74, 1.60)	1.08 (0.72, 1.64)
Recombinant [19,20] (SP-C)	3	237	239	0.36	0	0.84	0.99 (0.74, 1.32)	-
Recombinant + bovine [18-20] (SP-B and SP-C)	4	253	258	5.52	0.46	0.14	1.00 (0.69, 1.46)	0.87 (0.48, 1.58)

^a^In the studies by Weg and colleagues [16], Gregory and colleagues [18], and Spragg and colleagues [19], those patients who received a comparable surfactant dose (the higher dose) were used for the pooled analysis.

**Table 4:** Jadad scoring items and allocation concealment of each study eligible for meta-analysis

	Weg and colleagues, 1994 [16]	Anzueto and colleagues, 1996 [17]	Gregory and colleagues, 1997 [18]	Spragg and colleagues, 2003 [19]	Spragg and colleagues, 2004 [20]
Jadad scoring items					
Was the study randomized?	Yes	Yes	Yes	Yes	Yes
Was the randomization method described and appropriate?	Yes	Yes	No	Yes	No
Was the study described as double-blind?	Yes	Yes	No	No	Yes
Was the method of blinding described and appropriate	Yes	Yes	No	No	Yes
Was there a description of withdrawals and dropouts?	Yes	Yes	Yes	No	Yes
Inappropriate method of randomization?	No	No	No	No	No
Inappropriate method of blinding?	No	No	No	No	No
Allocation concealment	Central office provided randomization assignment to study sites	Independent central facility provided randomization assignment to study sites	Not clearly stated	Centralized facility provided randomization assignment to study sites	Not clearly stated
